# Neuroinflammation and Behavior in HIV-1 Transgenic Rats Exposed to Chronic Adolescent Stress

**DOI:** 10.3389/fpsyt.2016.00102

**Published:** 2016-06-20

**Authors:** Sydney A. Rowson, Constance S. Harrell, Mandakh Bekhbat, Apoorva Gangavelli, Matthew J. Wu, Sean D. Kelly, Renuka Reddy, Gretchen N. Neigh

**Affiliations:** ^1^Molecular and Systems Pharmacology Graduate Studies Program, Emory University, Atlanta, GA, USA; ^2^Neuroscience Graduate Studies Program, Emory University, Atlanta, GA, USA; ^3^Neuroscience and Behavioral Biology, Emory College, Atlanta, GA, USA; ^4^Department of Physiology, Emory University, Atlanta, GA, USA; ^5^Department of Psychiatry and Behavioral Science, Emory University, Atlanta, GA, USA

**Keywords:** sex differences, stress, HIV, neuroinflammation, anxiety, microglia

## Abstract

Highly active antiretroviral therapy (HAART) has improved prognosis for people living with HIV (PLWH) and dramatically reduced the incidence of AIDS. However, even when viral load is controlled, PLWH develop psychiatric and neurological disorders more frequently than those living without HIV. Adolescents with HIV are particularly susceptible to the development of psychiatric illnesses and neurocognitive impairments. While both psychiatric and neurocognitive disorders have been found to be exacerbated by stress, the extent to which chronic stress and HIV-1 viral proteins interact to impact behavior and relevant neuroinflammatory processes is unknown. Determination of the individual contributions of stress and HIV to neuropsychiatric disorders is heavily confounded in humans. In order to isolate the influence of HIV-1 proteins and chronic stress on behavior and neuroinflammation, we employed the HIV-1 transgenic (Tg) rat model, which expresses HIV-1 proteins with a *gag* and *pol* deletion, allowing for viral protein expression without viral replication. This Tg line has been characterized as a model of HAART-controlled HIV-1 infection due to the lack of viral replication but continued presence of HIV-1 proteins. We exposed male and female adolescent HIV-1 Tg rats to a mixed-modality chronic stress paradigm consisting of isolation, social defeat and restraint, and assessed behavior, cerebral vascularization, and neuroinflammatory endpoints. Stress, sex, and presence of the HIV-1 transgene impacted weight gain in adolescent rats. Female HIV-1 Tg rats showed decreases in central tendency during the light cycle in the open field regardless of stress exposure. Both male and female HIV-1 Tg rats exhibited decreased investigative behavior in the novel object recognition task, but no memory impairments. Adolescent stress had no effect on the tested behaviors. Microglia in female HIV-1 Tg rats exhibited a hyper-ramified structure, and gene expression of complement factor B was increased in the hippocampus. In addition, adolescent stress exposure increased microglial branching and junctions in female wild-type rats without causing any additional increase in HIV-1 rats. These data suggest that the presence of HIV-1 proteins during development leads to alterations in behavioral and neuroinflammatory endpoints that are not further impacted by concurrent chronic adolescent stress.

## Introduction

Following the advent of highly active antiretroviral therapy (HAART) in the treatment of HIV-1 infection, the life expectancy for people living with HIV (PLWH) has greatly increased (1). However, with increasing life expectancy among PLWH, complications not traditionally associated with HIV infection have emerged (1). These comorbidities include increased incidence of affective disorders, HIV-1-associated neurocognitive disorders (HAND), and non-AIDS-associated dementia and can occur even in individuals with HAART-controlled viral load. In fact, as many as 52% of PLWH experience some form of neuropsychological impairment (2). PLWH are at a greater risk of developing a mood disorder (3), and individuals with HAND are less likely to adhere to life-saving treatment regimens (4), increasing the risk of death (5). As many as 60% of the adolescents living with HIV develop psychiatric illnesses (6, 7) or neurocognitive impairments (8). These adolescents are at high risk of unsafe behaviors (9), but the mechanisms underlying this sensitivity to neuropsychiatric disease are unknown. Furthermore, the risks may be particularly high for HIV-infected females, given the established increased risk for affective disorders in women over men even in the absence of HIV (10, 11).

While stigma or distress associated with HIV-positive status has been suggested to contribute to neurocognitive impairment and the increased prevalence of mood disorders among PLWH, recent studies suggest that these behavioral pathologies are also mediated by biological mechanisms that extend beyond the direct impact of psychosocial stress associated with being HIV-positive (12–16). These studies have examined HIV-associated alterations in behavior and their underlying biological mechanisms, many of which involve neuroinflammatory processes. However, due to the difficulty of disentangling the impact of HIV-1 viral proteins and psychosocial stress in PLWH, the potential interaction between chronic stress and HIV-1 viral proteins remains incompletely understood. Using the HIV-1 transgenic (Tg) rat model, we sought to examine the contributions of developmental exposure to HIV-1 proteins and chronic adolescent stress in order to better understand the extent to which HIV and stress interact in adolescents with HIV.

The HIV-1 Tg rat expresses all but two of the genes contained in the HIV-1 virus, which allows expression of functional viral proteins without active viral replication (17). This non-replicating HIV-1 Tg line in the rat has been characterized as a useful tool to model comorbidities experienced by PLWH receiving HAART (18) as well as a model for other childhood HIV-1-associated disorders (19).

Here, we used the HIV-1 Tg rat to address the hypothesis that chronic adolescent stress exacerbates behavioral and neuroinflammatory impairments in HIV-1 Tg rats compared to wild-type (WT) controls in a sex-specific manner. We focused on inflammatory endpoints due to their capacity to modulate affective and cognitive behavior as well as the profound immune alterations seen in HIV-1 infection. We assessed microglial morphology in females as well as gene expression of complement factor B and lipocalin-2, two inflammatory proteins that may also be involved in neuronal structure and the impairments observed in PLWH, in non-stressed female rats. Collectively, the data presented suggest that females are more susceptible to the behavioral effects of HIV-1 protein expression during development than males and that HIV-1 proteins mirror, but do not interact with, the neuroinflammatory impact of chronic adolescent stress in females.

## Materials and Methods

### Animals

Wild-type and HIV-1 Tg male and female rats were bred on-site from Tg male breeders and WT female Fisher 344/NHsd dams purchased from Harlan Laboratories (Indianapolis, IN, USA). The HIV-1 Tg rat, originally described by Reid et al. (17), was derived from the Fisher 344/NHsd Sprague–Dawley line. HIV-1 Tg rats show clinical manifestations and pathology resembling HIV, including respiratory problems, neurological changes, cataracts, nephropathy, muscle atrophy, and altered affective-type behavior (15, 17, 19, 20). The studies presented here were completed by postnatal day (PND) 55 and thus occurred prior to the onset of previously documented pathology. Offspring were group-housed with siblings (2–3 rats per cage) after weaning. Male and female rats were used for all behavioral analyses, while female rats were used for histological and gene expression assessments. Animals were kept in an AAALAC-approved temperature- and humidity-controlled vivarium and maintained on a reverse 14:10 light:dark cycle. Food and water were available *ad libitum*. For these studies, litters were split among all groups such that no more than two pups from a litter were in any one group. This measure was taken in order to avoid litter effects. Chronic mixed-modality adolescent stress took place from PND37 to PND48; behavioral assessments were performed from PND49 to PND53, and rats were euthanized between PND54 and PND55. All experiments were performed in accordance with the Institutional Animal Care and Use Committee of Emory University and the National Institutes of Health *Guide for the Care and Use of Laboratory Animals*.

### Chronic Mixed-Modality Stress

A subset of each cohort was submitted to chronic mixed-modality adolescent stress (WT-male-control *n* = 8, WT-male-stressed *n* = 9, WT-female-control *n* = 11, WT-female-stressed *n* = 10, Tg-male-control *n* = 11, Tg-male-stressed *n* = 11, Tg-female-control *n* = 10, and Tg-female-stressed *n* = 9). This mixed-modality stress paradigm has been previously described and shown to elicit both short-term and lasting behavioral and physiological changes in Wistar rats (21–24). Consistent with the previously established paradigm, animals receiving stress were individually housed at PND36 and randomly exposed to interspersed days of social defeat and restraint stress for 12 days (PND37–PND48). Stressed animals were individually housed throughout the duration of behavioral testing. Non-stressed animals remained pair-housed throughout the entire study and encountered daily handling and cage movement to control for the stress paradigm. Rats were weighed on isolation day and twice throughout stress.

The 6 days of social defeat stress were performed during the light phase of the light:dark cycle and took place in the home cage of a mature, territorial, Long–Evans rat. All experimental animals were paired with a same-sex resident Long–Evans rat, and female resident Long–Evans rats were ovariectomized to control for estrous cycle-dependent variations in aggressive behavior. Each experimental animal was placed in the home cage of the resident and separated by a barrier permeable to sound and scent; after 2 min, the barrier was lifted. After the experimental animal was aggressed by the resident up to five times on the first day, three times on the second day, and once each day thereafter, or after 5 min of interaction, the barrier was replaced. This separation continued for 25 min, after which point the experimental animal was returned to its home cage. Pairings were randomly assigned daily to prevent stabilization of a dominance hierarchy.

For the 6 days of restraint stress, animals were placed in a clear acrylic rat restraint (Braintree Scientific, Inc., Braintree, MA, USA) for 60 min during the light phase. These restraints prevented head-to-tail turns but did not compress the rat.

### Behavioral Testing

Behavioral testing took place from PND49 to PND53, after the completion of the stress paradigm, and consisted of open-field testing and novel object testing. The behavioral testing was completed in six cohorts across a 4-month span of time with all groups represented equally in each cohort. The length of testing for any one cohort was 5 days. Sample sizes are detailed in Table 1.

**Table 1:** **Sample sizes for each of the behavioral tests (open-field and novel object recognition task), animal weight data, and molecular analyses are detailed**.

Test	F WT NS	F Tg NS	M WT NS	M Tg NS	F WT S	F Tg S	M WT S	M Tg S
Novel object	9	7	8	9	10	4	8	11
Open field	11	10	7	11	10	9	8	9
Body weight over stress	11	10	8	11	10	9	8	11
Terminal weight	11	10	8	9	9	9	6	11
Microglial analysis	3	3	N/A	N/A	3	3	N/A	N/A
Lcn2 gene expression	7	10	N/A	N/A	N/A	N/A	N/A	N/A
Cfb gene expression	10	11	N/A	N/A	N/A	N/A	N/A	N/A

*Animals used in behavior analysis were included in weight analysis. M, male; F, female; NS, no stress; S, stress; WT, wild type; Tg, transgenic*.

#### Open-field Testing

Measurement of locomotor activity and spatial preference during the open-field test can be used as an assessment of both physiological capacity as well as anxiety-like behavior (25). Animals were assessed in the open field during the light phase under white light (between 8:00 and 11:00 a.m.) on PND49 and during the dark phase under red lighting (between 3:00 and 6:00 p.m.) on PND50. For both tests, each rat was placed into a 90 cm × 90 cm box and allowed to explore freely for 10 min, after which time the rat was placed back in its home cage, and the open-field arena was cleaned thoroughly with 70% ethanol. One rat was tested at a time. All behaviors in both the light and dark phase testing were recorded by a video camera that was connected to an automated behavior analysis system and analyzed by research assistant blind to experimental group (CleverSys, Inc., Reston, VA, USA). Four animals (Tg-male-stressed *n* = 2, WT-male-control *n* = 1, and WT-male-stressed *n* = 1) were excluded from open-field analysis due to a procedural error.

#### Novel Object Testing

The novel object test is used to assess object recognition memory, a task that is dependent on hippocampal function (26). Training and testing occurred during the light phase between 8:00 a.m. and 12:00 p.m., and two animals were tested simultaneously, individually in separate chambers. The chamber used for the open-field test was subdivided into two 90 cm × 45 cm arenas. On PND51, rats were habituated to the smaller arena with a 10-min exploration period. On PND52 and 53, rats were exposed to the no-delay and the hour-delay tasks in a counterbalanced order. In the no-delay task, the rat was placed into the arena with two identical objects (“familiar objects”) and allowed to explore for 15 min. After this period, the rat was briefly returned to its home cage; the objects and arena were quickly cleaned with 70% ethanol, and one of the objects was replaced with a novel object. The rat was returned to the arena and allowed to explore the objects for 5 min. All behaviors during this period were recorded by a video camera that was connected to an automated behavior analysis system and analyzed by a research assistant blind to experimental group (CleverSys, Inc., Reston, VA, USA). The hour-delay task was identical to the no-delay task but used different novel and familiar objects, and the rat was returned to its home cage for a 1-h delay before undergoing the 5-min recorded encounter. Several rats were excluded from novel object behavioral analysis due to a procedural error (WT-female-control *n* = 2, WT-male-stressed *n* = 1, Tg-male-control *n* = 2, Tg-female-control *n* = 2, and Tg-female-stressed *n* = 5). Difference in time spent sniffing novel vs. familiar object was calculated by taking the absolute value of the difference in time spent sniffing the novel and familiar objects. One animal (Tg-female-control) was identified as an outlier *via* Grubbs’ test (α = 0.05) and was removed from novel object analysis.

### Tissue Collection

On PND54–55, all animals used for behavioral tests and stereology were euthanized with Euthasol® (a combination of pentobarbital sodium and phenytoin sodium), perfused for 2 min with ice cold saline, and then perfused with 4% paraformaldehyde for 10 min. Estrous cycle was not monitored in females. Brains were removed and postfixed in 4% paraformaldehyde and cryoprotected in 30% sucrose for 24 h before freezing. Female WT (*n* = 11) and HIV-1 Tg (*n* = 11) non-stressed animals that did not undergo behavioral testing were euthanized *via* rapid decapitation for quantitative real-time polymerase chain reaction (qPCR) analysis. Brains were flash frozen on dry-ice and stored at −80°C until later analysis. In addition, adrenal glands, uteri, and testes were collected, weighed, and normalized to terminal weights. Percentage weight change over stress was calculated by dividing the difference between day 10 stress weight and isolation weight by the isolation weight. Normalized adrenal and reproductive weights were calculated by dividing each rat’s adrenal or reproductive weight (milligrams) by its terminal body weight (grams). All weight analyses were performed with animals that underwent behavioral testing.

### Immunohistochemistry and Stereology

Brains from female rats were sectioned at 40 or 50 μm on a cryostat.

#### IBA-1 Immunohistochemistry

Sections from female WT-control, WT-stress, Tg-control, and Tg-stress (*n* = 3 per group) rats encompassing the entire rostro–caudal axis of the brain were stained for ionized calcium-binding adaptor molecule-1 (IBA-1) [four sections per animal, section sampling fraction (ssf) = 1/12]. After washing in TBS, sections were blocked in a 10 μg/ml avidin solution for 1 h at 4°C. Sections were washed again and then incubated in rabbit IBA-1 (1:1000, 019-19741, WAKO, Richmond, VA, USA) in a 50 μg/ml biotin solution overnight at 4°C. Sections were then washed and incubated in biotinylated goat anti-rabbit IgG (BA-1000, Vector Labs, Burlingame, CA, USA) for 1 h at 4°C before washing and incubating with the Vectastain Elite ABC kit (Vector Labs, Burlingame, CA, USA). The stain was then visualized with diaminobenzidine (SigmaFast 3,3′-diaminobenzidine tablets, Sigma–Aldrich, St. Louis, MO, USA), and sections were counterstained in cresyl violet. The optical fractionator probe was used to estimate the population of microglia in a single hemisphere of the hippocampus.

#### Microglial Morphology Analysis

Right hippocampal regions from four sections (three from one animal due to tissue integrity) from each animal were stitched together at 40× magnification (StereoInvestigator). Thirty cells were chosen at random per section, converted to 8-bit, adjusted for brightness, and cleaned with a Gaussian filter. Images were then converted to binary and skeletonized in ImageJ (National Institute of Health, version 1.49). The AnalyzeSkeleton plug-in was used to assess the number of junctions and average branch length for each microglia. Microglial properties (number of junctions, number of branches, maximum and average branch length) were averaged for each animal.

#### Cerebrovascular Immunohistochemistry

Sections from female rats were incubated with 1% H_2_O_2_ for 20 min at 4°C followed by blocking with normal goat serum (NGS) (3%) (Vector Labs, Burlingame, CA, USA), Triton X-100 (0.5%) (Sigma–Aldrich, St. Louis, MO, USA), and phosphate-buffered saline (PBS). Sections were then incubated with primary antibody mouse anti-rat endothelial cell antigen-1 [RECA-1 (0.5%) (Bio-Rad Labs, Berkeley, CA, USA)] overnight at 4°C. The following day, sections were incubated with biotinylated anti-mouse IgG secondary antibody (0.5%) (Vector Labs, Burlingame, CA, USA). Sections were then incubated with streptavidin–peroxidase–HRP. The stain was visualized with diaminobenzidine. Sections were mounted on slides, dried overnight, and coverslipped with Permount (Fisher Scientific, Pittsburgh, PA, USA).

#### Blood Vessel Length and Density

To minimize bias, stereology was conducted by a single experimenter blind to experimental conditions. To determine the location of the prefrontal cortex (PFC) (WT *n* = 3, Tg *n* = 5), amygdala (WT *n* = 5, Tg *n* = 6), and hippocampus (WT *n* = 5, Tg *n* = 6) in each section, a rat atlas (27) was used as a guide to trace contours under 2× bright-field illumination. Three sections were used per animal for analysis. After tracing contours, sections were sampled under a 60× oil immersion objective. Estimates were taken using the Stereo Investigator system with the Spaceballs probe (MicroBrightField, Inc., Colchester, VT, USA). The following parameters were used in the Spaceballs probe for 40 and 50 μm sections analyzing the PFC: grid size *X* = 230 μm, grid size *Y* = 315 μm, guard zone space = fixed distance, Spaceball radius = 12 μm, and use hemisphere = true. Additionally, the following parameters were used for sections analyzing the hippocampus and amygdala: grid size *X* = 275 μm, grid size *Y* = 360 μm, guard zone space = fixed distance, Spaceball radius = 12 μm, and use hemisphere = true. These parameters resulted in a coefficient of error of ≤0.1.

### Quantitative Real-Time Polymerase Chain Reaction

The PFC and hippocampus were dissected from female WT (*n* = 11) and HIV-1 Tg (*n* = 11) rats, and RNA was extracted with the TRIzol method (Invitrogen) using the RNeasy Mini Kit (Qiagen). RNA was reverse-transcribed using the High Capacity cDNA Reverse Transcription Kit (Applied Biosystems). cDNA was standardized using a Pico Green Assay (Invitrogen). TaqMan Gene Expression Mastermix and TaqMan Gene Expression Assays for *Hprt1* (Rn01527840_m1) and *Cfb* (Rn01526084_g1) were used. qPCR was performed on the Applied Biosystems 7900HT Sequence Detection System. Fold change in gene expression was calculated according to the 2^−ΔΔCt^ method using the *Hprt1* housekeeping gene. The targets were run in triplicate, and the cutoff for coefficient of variance (CV) was 4%. One WT animal was excluded from *Cfb* analysis because its CV value exceeded the 4% cutoff.

Gene expression of *Lcn2* (female WT *n* = 7, Tg *n* = 10) was assessed using primers from Integrated DNA Technologies (San Diego, CA, USA). Forward: GATTCGTCAGCTTTGCCAAGT and reverse: CATTGGTCGGTGGGAACAG. Absolute Blue qPCR SYBR Green ROX Mix (Thermo Scientific, Wilmington, DE, USA) was used to perform qPCR. Hippocampal gene expression of *Lcn2* was normalized to the geometric mean of *Hprt* and *B2m*, whereas PFC expression of *Lcn2* was normalized to the geometric mean of *B-actin*, *Hprt1*, *B2m*, and *Ldha*.

### Statistical Analysis

Three-way analysis of variance (ANOVA) in R (version 3.2.1) was used to assess statistical significance in behavioral tests, percentage weight gain, and normalized adrenal measures. The analysis factors in three-way analysis of percentage weight gain and normalized adrenal weight were all between-subjects and included sex, stress (control, adolescent stress), and genotype (WT, HIV-1 Tg). The analysis factors in three-way analyses of behavior were the between-subjects factors of stress (control, adolescent stress) and genotype (WT, HIV-1 Tg) with the repeated factor of time (light and dark in the open-field test or no delay and hour delay in the novel object test). Because of the added factor of time, sexes were assessed separately. If no main effect was present, the non-significant factor was removed from *post hoc* assessment leading to a *t*-test assessment. Two-way ANOVAs in GraphPad Prism (version 6) were used to assess group differences for reproductive weight, microglial population, and microglial morphology. The factors in two-way analyses were stress and genotype. Student’s *t*-test was performed to assess statistical significance of number of microglial branches and number of microglial junctions between the WT control group and the Tg control group. A student’s *t*-test was used to assess statistical significance for gene expression data. Each gene and region was compared to its WT control. All tests were performed with α = 0.05, and all significant main effects and interactions are reported in the section “Results.”

## Results

### Both Chronic Adolescent Stress and HIV-1 Proteins Reduced Weight Gain

**Figure 1:**
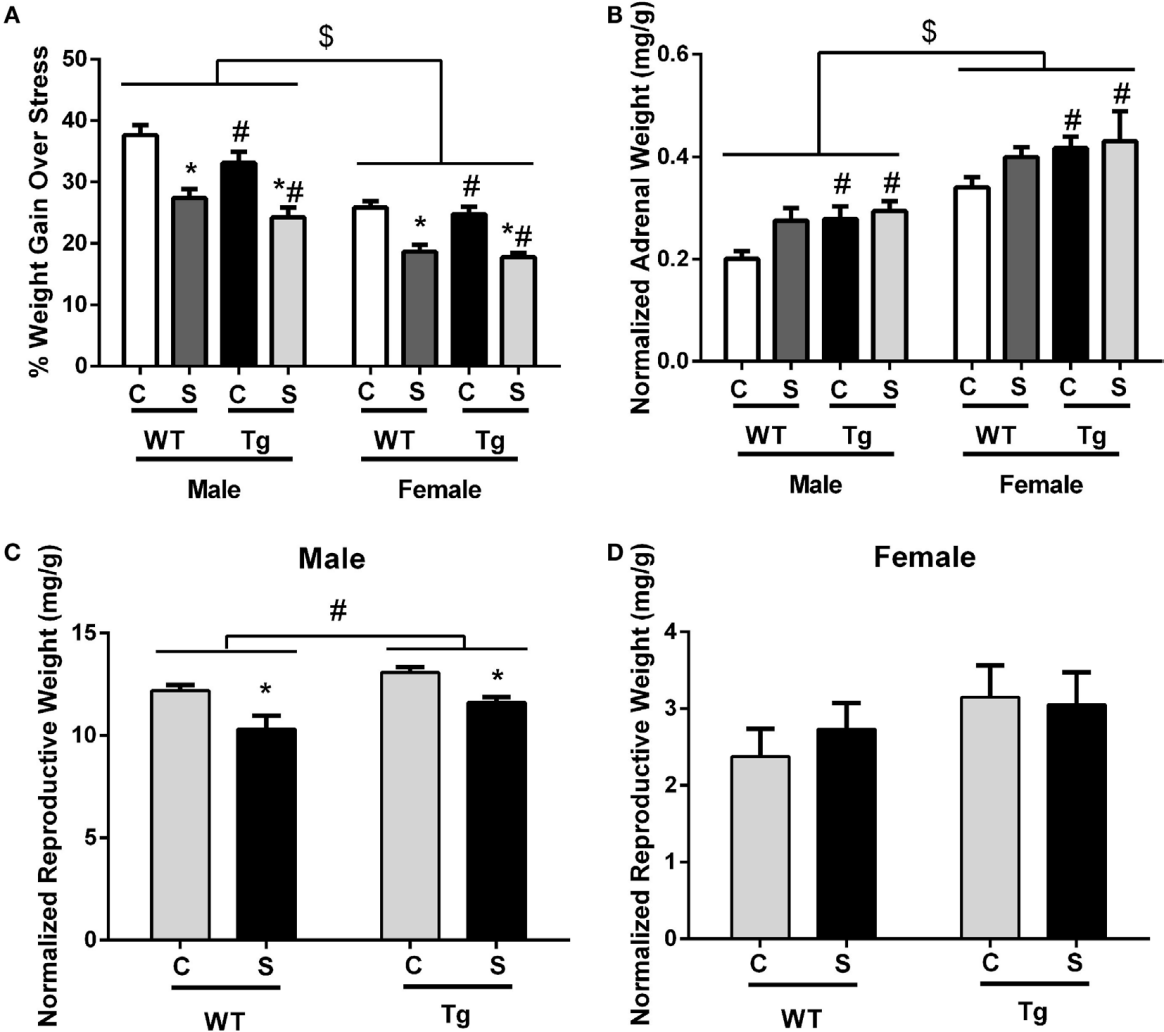
**Chronic adolescent stress reduced weight gain from PND36 to PND46**. Females and HIV-1 Tg rats also gained less weight during stress **(A)**. Terminal adrenal weight was normalized to terminal body weight, and normalized adrenal weight was increased in HIV-1 Tg rats and in females **(B)**. Normalized reproductive weight was decreased in males exposed to adolescent stress but increased with HIV-1 transgene **(C)**. Normalized reproductive weight in females was unchanged by HIV-1 transgene or stress **(D)**. Data are presented as mean ± SEM. # denotes a main effect of HIV-1 Tg genotype, *Denotes a main effect of stress, and $ denotes a main effect of sex. α = 0.05.

Exposure to chronic adolescent stress reduced weight gain in stressed animals [*F*_(1,70)_ = 70.91, *p* < 0.01] (Figure 1A). Females [*F*_(1,70)_ = 79.21, *p* < 0.01] and HIV-1 Tg rats [*F*_(1,70)_ = 5.80, *p* = 0.02] also gained less weight during stress as compared to males and WT rats. However, terminal weight was unchanged by stress [*F*_(1,65)_ = 1.72, *p* > 0.05]. HIV-1 Tg rats weighed less at collection than WT rats [*F*_(1,65)_ = 150.88, *p* = 0.01]. Female rats weighed less than males [*F*_(1,65)_ = 199.81, *p* < 0.01] (Table 2). Normalized adrenal weight was higher in females [*F*_(1,65)_ = 44.60, *p* < 0.01] and HIV-1 Tg rats [*F*_(1,65)_ = 6.53, *p* = 0.01] (Figure 1B). Normalized reproductive weight was analyzed separately between the sexes due to the distinct differences in sex organs. Stress [*F*_(1,31)_ = 6.87, *p* = 0.01] and HIV-1 transgene [*F*_(1,31)_ = 16.09, *p* < 0.01] increased male reproductive weight (Figure 1C). Neither stress [*F*_(1,34)_ = 2.05, *p* > 0.05] nor HIV-1 genotype [*F*_(1,34)_ = 0.12, *p* > 0.05] impacted normalized reproductive weight in females (Figure 1D). Details of statistical analyses are detailed in Table 3.

**Table 2:** **Terminal weight (in grams) of rats that were exposed to chronic adolescent stress and behavioral testing**.

	Wild type		HIV-1 transgenic	
	
	No stress	Stress	No stress	Stress
Male	176.0 ± 4.56 g	168.0 ± 7.32 g	135.7 ± 4.59 g^b^	138.5 ± 2.81 g^b^
Female	135.2 ± 2.34 g^a^	129.9 ± 3.12 g^a^	107.3 ± 1.56 g^a,b^	103.1 ± 3.04 g^a,b^

*Mean ± SEM are shown. HIV-1 Tg rats weighed less than WT rats [*F*_(1,65)_ = 150.88, *p* < 0.01]. Females weighed less than males [*F*_(1,65)_ = 199.81, *p* < 0.01]*.

*^a^Denotes a main effect of sex*.

*^b^denotes a main effect of HIV-1 genotype*.

**Table 3:** ***F*-statistics and *p*-values for all main effects and interactions are shown for weight analysis using a three-way ANOVA, between factors (sex, HIV-1 genotype, time), or a two-way ANOVA, between factors (HIV-1 genotype and stress)**.

	Sex	HIV-1 genotype	Stress	Sex × HIV-1 genotype	Sex × stress	Stress × HIV-1 genotype	Sex × stress ×HIV-1 genotype
Weight over stress (1A)	*F_(1,70)_ = 79.2, *p* < 0.01	*F_(1,70)_ = 5.80, *p* = 0.02	*F_(1,70)_ = 70.91, *p* < 0.01	*F*_(1,70)_ = 2.11, *p* > 0.05	*F*_(1,70)_ = 1.54, *p* > 0.05	*F*_(1,70)_ = 0.16, *p* > 0.05	*F*_(1,70)_ = 0.09, *p* > 0.05
Adrenal weight (1B)	*F_(1,65)_ = 44.60, *p* < 0.01	*F_(1,65)_ = 6.53, *p* = 0.01	*F*_(1,65)_ = 3.66, *p* > 0.05	*F*_(1,65)_ = 0.02, *p* > 0.05	*F*_(1,65)_ = 0.04, *p* > 0.05	*F*_(1,65)_ = 1.66, *p* > 0.05	*F*_(1,65)_ = 0.03, *p* > 0.05
Male reproductive weight (1C)		*F_(1,31)_ = 16.09, *p* < 0.01	*F_(1,31)_ = 6.87, *p* = 0.01			*F*_(1,31)_ = 0.25, *p* > 0.05	
Female reproductive weight (1D)		*F*_(1,34)_ = 0.12, *p* > 0.05	*F*_(1,34)_ = 2.05, *p* > 0.05			*F*_(1,34)_ = 0.35, *p* > 0.05	
Terminal weight (Table 2)	*F_(1,65)_ = 199.8, *p* < 0.01	*F_(1,65)_ = 150.9, *p* < 0.01	*F*_(1,65)_ = 1.72, *p* > 0.05	*F*_(1,65)_ = 2.41, *p* > 0.05	*F*_(1,65)_ = 0.27, *p* > 0.05	*F*_(1,65)_ = 1.22, *p* > 0.05	*F*_(1,65)_ = 0.93, *p* > 0.05

*Indicates a significant main effect or interaction. Blank boxes indicate an absence of a particular factor from analysis. The figure or table number is included in parentheses following the description of each metric. α = 0.05.

### Female HIV-1 Tg Rats had Reduced Central Tendency in the Open-field Maze

**Figure 2:**
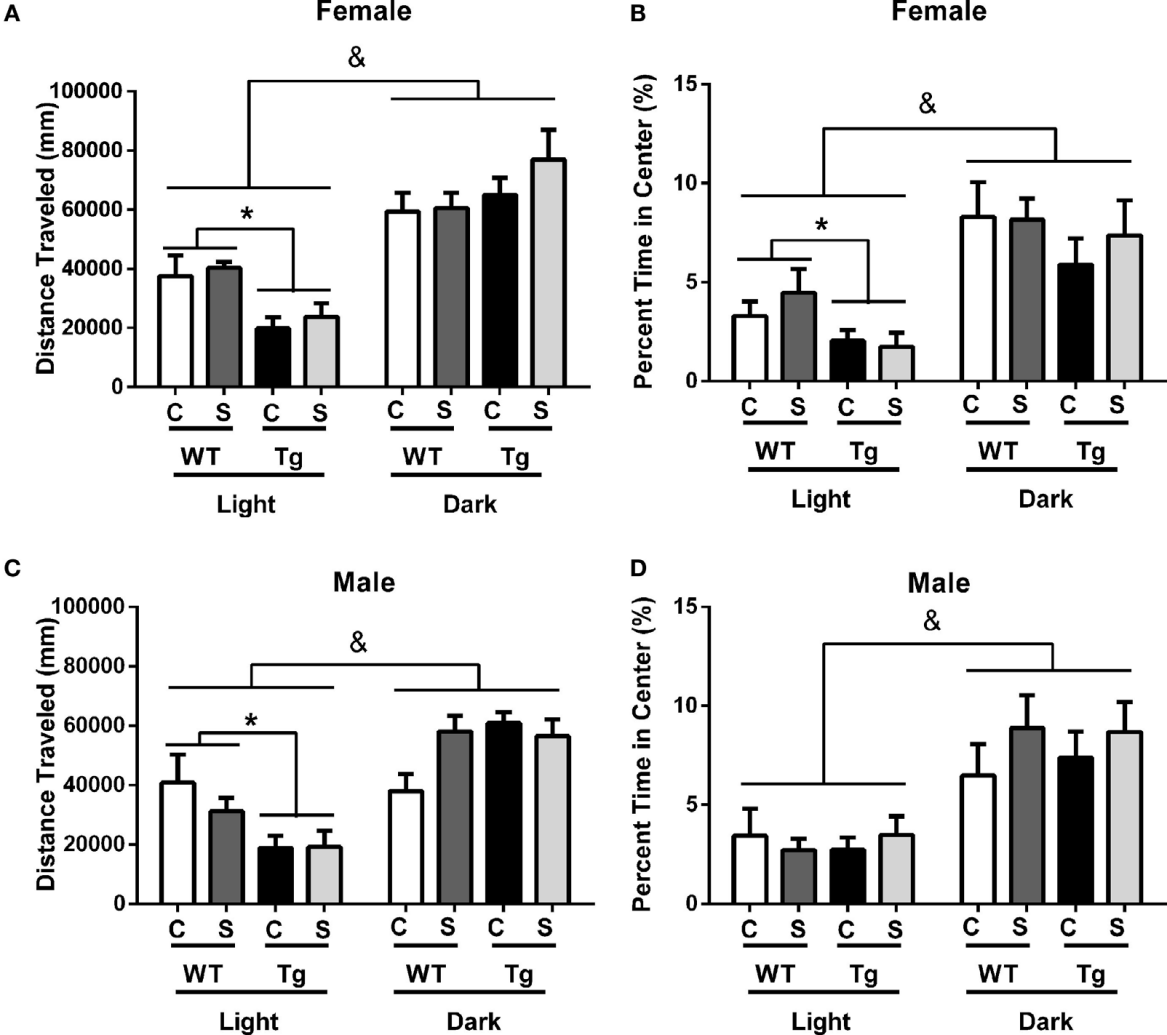
**HIV-1 Tg and WT rats were placed in an open-field maze during the rats’ light and dark cycles**. The distance traveled and the percentage of time the rats spent in the center of the maze were measured. HIV-1 Tg rats traveled a decreased distance compared to WT controls in the light cycle **(A)**. Male **(C)** and female **(A)** rats traveled an increased distance in the dark cycle compared to the light cycle. The percentage of time rats spent in the center was unaffected by stress or transgene in males **(D)**. Female Tg rats spent a decreased percentage of time in the center compared to WT female rats during the light cycle **(B)**. Male **(D)** and female **(B)** rats spent an increased percentage of the time in the center during the dark cycle. Data are presented as mean ± SEM. & denotes the main effect of time. *Denotes a main effect in a *t*-test collapsed across stress. α = 0.05.

As expected for a nocturnal species, male [*F*_(1,31)_ = 56.86, *p* < 0.01] and female [*F*_(1,36)_ = 52.55, *p* < 0.01] rats, regardless of stress exposure or genotype, traveled greater distance in the open-field maze during the dark cycle than during the light cycle. Stress did not independently impact locomotor activity [*F*_(1,36)_ = 1.78, *p* > 0.05] or percentage time in center [*F*_(1,36)_ = 0.38, *p* > 0.05] for female rats of either genotype. *Post hoc* assessment demonstrated that female HIV-1 Tg rats exhibited decreased locomotor activity compared to female WT rats in the light cycle (*t*_38_ = 3.62, *p* < 0.001) (Figure 2A) and that female HIV-1 rats spent a decreased time in the center of the open-field maze compared to female WT rats during the light cycle [*t*_(1,38)_ = 2.35, *p* = 0.024] (Figure 2B). Male HIV-1 rats also exhibited decreased locomotor activity compared to male WT rats in the light cycle (*t*_33_ = 2.93, *p* < 0.01). Female [*F*_(1,36)_ = 29.35, *p* < 0.001] and male [*F*_(1,31)_ = 24.46, *p* < 0.001] rats also spent an increased percentage of time in the center of the open-field maze during the dark cycle, as compared to percentage time in center during the light cycle (Figures 2B,D). HIV-1 transgene and time of day interacted in males [*F*_(1,31)_ = 13.23, *p* < 0.001] and females [*F*_(1,36)_ = 8.68, *p* < 0.01] to impact the locomotor activity of males in the open-field maze. HIV-1 Tg genotype, stress, and sex [*F*_(1,31)_ = 5.11, *p* = 0.03] also interacted to impact the locomotor activity of males (Figure 2C) in the open-field maze. However, *post hoc* assessment did not attribute these main effects or interactions to differences between any two specific groups. All main effects and interactions are detailed in Table 4.

**Table 4:** ***F*-statistics and *p*-values for all main effects and interactions are shown for behavior analysis using a three-way ANOVA between factors (HIV-1 genotype, stress) and within factors (time)**.

	HIV-1 genotype	Stress	Time	Stress × time	HIV-1 genotype × time	Stress × HIV-1 genotype	Stress × HIV-1 genotype × time
Female distance traveled (2A)	*F*_(1,36)_ = 0.78, *p* > 0.05	*F*_(1,36)_ = 1.78, *p* > 0.05	*F_(1,36)_ = 52.5, *p* < 0.01	*F*_(1,36)_ = 0.10, *p* > 0.05	*F_(1,36)_ = 8.68, *p* < 0.01	*F*_(1,36)_ = 0.67, *p* > 0.05	*F*_(1,36)_ = 0.26, *p* > 0.05
Female percent time in center (2B)	*F*_(1,36)_ = 4.06, *p* > 0.05	*F*_(1,36)_ = 0.38, *p* > 0.05	*F_(1,36)_ = 29.3, *p* < 0.01	*F*_(1,36)_ < 0.01, *p* > 0.05	*F*_(1,36)_ = 0.03, *p* > 0.05	*F*_(1,36)_ < 0.01, *p* > 0.05	*F*_(1,36)_ = 0.86, *p* > 0.05
Male distance traveled (2C)	*F*_(1,31)_ = 0.66, *p* > 0.05	*F*_(1,31)_ = 0.08, *p* > 0.05	*F_(1,31)_ = 56.9, *p* < 0.01	*F*_(1,31)_ = 1.78, *p* > 0.05	*F_(1,31)_ = 13.2, *p* < 0.01	*F*_(1,31)_ = 0.87, *p* > 0.05	*F_(1,31)_ = 5.11, *p* = 0.03
Male percent time in center (2D)	*F*_(1,31)_ = 0.06, *p* > 0.05	*F*_(1,31)_ = 1.53, *p* > 0.05	*F_(1,31)_ = 24.5, *p* < 0.01	*F*_(1,31)_ = 0.72, *p* > 0.05	*F*_(1,31)_ = 0.03, *p* > 0.05	*F*_(1,31)_ = 0.01, *p* > 0.05	*F*_(1,31)_ = 0.42, *p* > 0.05
Female total time sniffing objects (3A)	*F_(1,26)_ = 6.78, *p* = 0.02	*F*_(1,26)_ = 0.02, *p* > 0.05	*F*_(1,26)_ = 1.53, *p* > 0.05	*F*_(1,26)_ = 0.06, *p* > 0.05	*F*_(1,26)_ = 0.61, *p* > 0.05	*F*_(1,26)_ = 2.07, *p* > 0.05	*F*_(1,26)_ = 0.13, *p* > 0.05
Female difference in sniffing time (3B)	*F_(1,26)_ = 5.39, *p* = 0.03	*F*_(1,26)_ = 0.90, *p* > 0.05	*F*_(1,26)_ = 1.25, *p* > 0.05	*F*_(1,26)_ = 0.82, *p* > 0.05	*F*_(1,26)_ = 0.45, *p* > 0.05	*F*_(1,26)_ = 3.28, *p* > 0.05	*F*_(1,26)_ = 0.45, *p* > 0.05
Male total time sniffing objects (3C)	*F_(1,32)_ = 7.50, *p* < 0.01	*F*_(1,32)_ = 0.31, *p* > 0.05	*F*_(1,32)_ < 0.01, *p* > 0.05	*F*_(1,32)_ = 0.05, *p* > 0.05	*F*_(1,32)_ = 0.45, *p* > 0.05	*F*_(1,32)_ = 0.02, *p* > 0.05	*F*_(1,32)_ = 1.04, *p* > 0.05
Male difference in sniffing time (3D)	*F*_(1,32)_ = 0.53, *p* > 0.05	*F*_(1,32)_ = 0.64, *p* > 0.05	*F*_(1,32)_ = 0.03, *p* > 0.05	*F*_(1,32)_ = 1.53, *p* > 0.05	*F*_(1,32)_ < 0.01, *p* > 0.05	*F*_(1,32)_ = 0.55, *p* > 0.05	*F_(1,32)_ = 5.17, *p* = 0.03

*Indicates a significant main effect or interaction. The figure or table number is included in parentheses following the description of each metric. α = 0.05.

### Female HIV-1 Tg Rats Exhibit Decreased Performance in Novel Object Recognition Task

**Figure 3:**
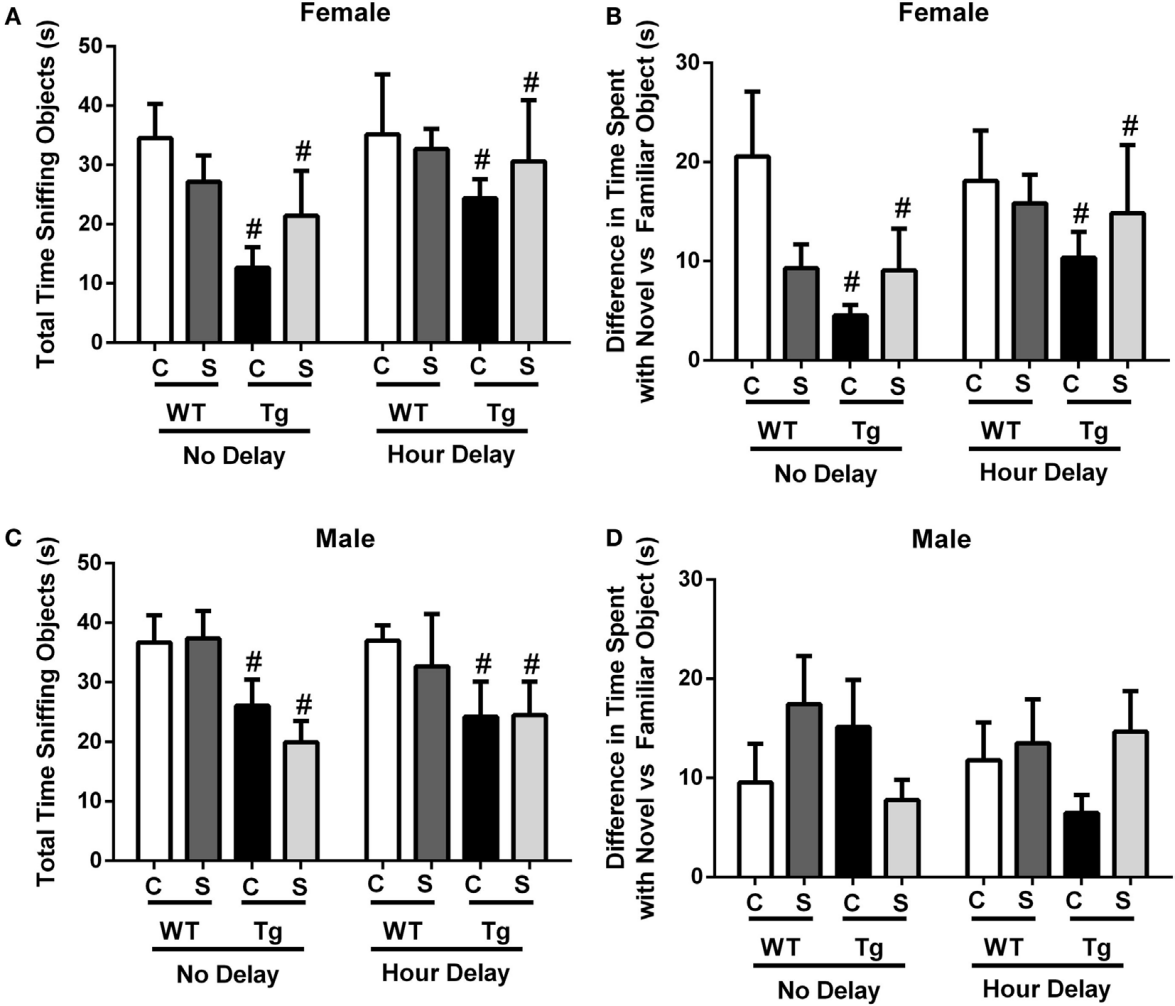
**Total time sniffing novel and familiar objects were measured in the novel object recognition task**. This task was performed with no delay or 1-h delay following exposure to familiar objects. Female HIV-1 Tg rats spent less time sniffing all objects **(A)**. Female Tg rats spent a reduced difference in time sniffing the novel vs. familiar object compared to female WT control rats **(B)**. Male HIV-1 Tg rats spent less time sniffing all objects **(C)**, and genotype, stress, and time interacted to impact the difference in time spent sniffing the novel vs. familiar object in males **(D)**. Data are presented as mean ± SEM. # denotes a main effect of HIV-1 Tg genotype. α = 0.05.

The novel object recognition task was performed with no delay or 1-h delay from exposure to familiar objects. Stress did not impact total time sniffing object in males [*F*_(1,32)_ = 0.31, *p* > 0.05] or females [*F*_(1,26)_ = 0.02, *p* > 0.05]. Time (no delay vs. hour delay) [*F*_(1,26)_ = 1.53, *p* > 0.05] had no impact on total time sniffing objects in females (Figure 3A). Female HIV-1 Tg rats spent less time sniffing all objects compared to WT females at each time point [*F*_(1,26)_ = 6.78, *p* = 0.02] (Figure 2A), and HIV-1 Tg females exhibited a reduced difference in time sniffing the novel vs. the familiar object compared to female WT rats [*F*_(1,26)_ = 5.39, *p* = 0.03] (Figure 3B). Male HIV-1 Tg rats spent a reduced total time sniffing all objects [*F*_(1,32)_ = 7.5, *p* < 0.01] (Figure 3C). Stress, genotype, and time interacted [*F*_(1,32)_ = 5.17, *p* = 0.03] to impact the difference in time spent sniffing the novel vs. familiar object in males (Figure 3D). This difference was not attributable to a particular group difference.

### HIV-1 Tg Rats Exhibit Enhanced Ramification in Hippocampal Microglia

**Figure 4:**
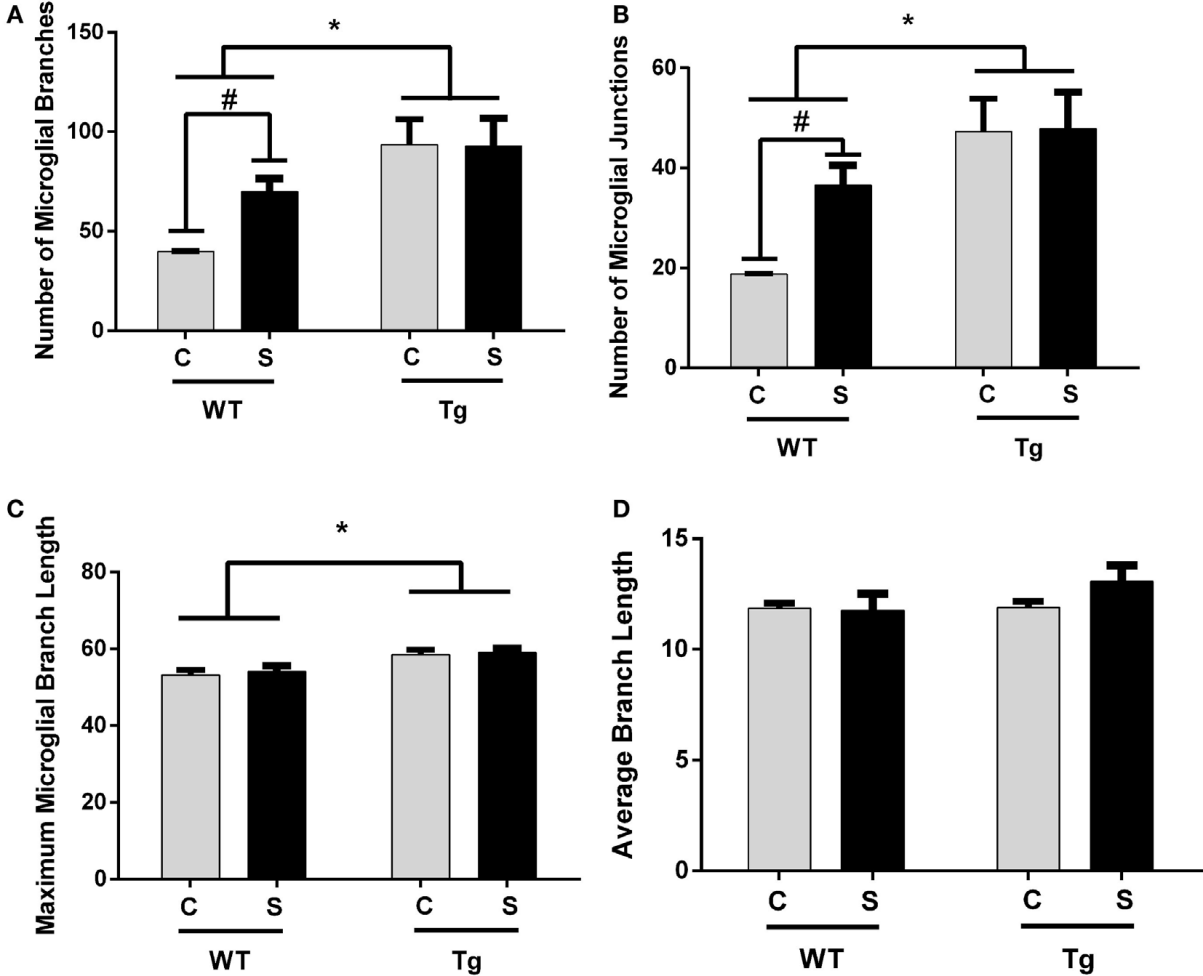
**Morphology of IBA-1-stained microglia was assessed using the AnalyzeSkeleton function in ImageJ**. Microglia in female HIV-1 transgenic rats exhibited enhanced ramification characterized by an increased number of branches **(A)**, junctions **(B)**, and maximum branch length **(C)**. Stress did not impact microglial morphology, and the average branch length was unchanged in any group **(D)**. Data are presented as mean ± SEM. *Denotes a main effect of HIV-1 transgene. # denotes significant effect of stress in an *a priori t*-test. α = 0.05.

**Figure 5:**
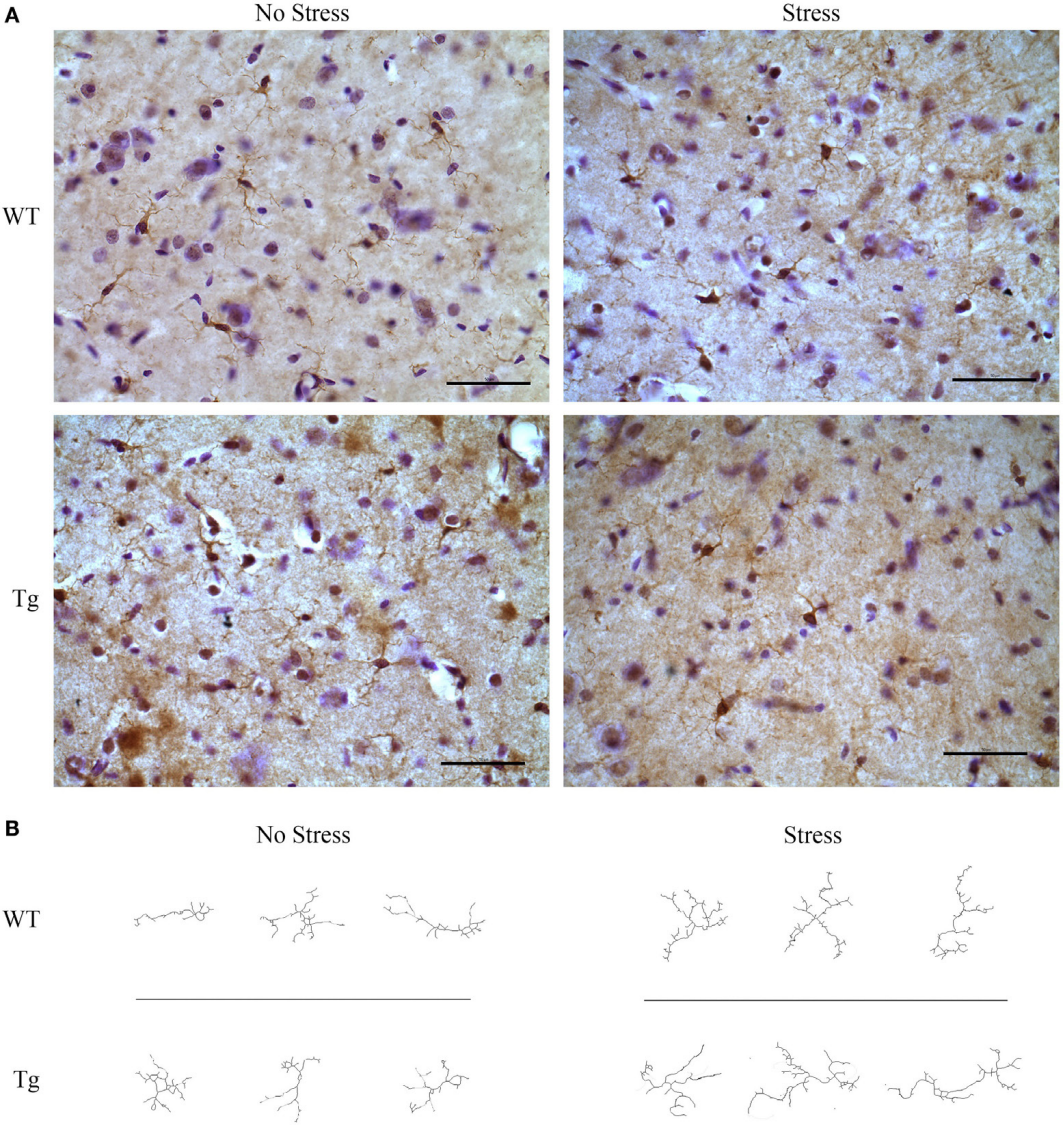
**HIV-1 transgenic and wild-type rats were exposed to chronic adolescent stress or left non-stressed during adolescence**. Brains were sectioned at 40 μm and stained for IBA-1 and visualized with diaminobenzidine. Representative images of the hippocampus for HIV-1 Tg, WT, stressed, and non-stressed rats are shown **(A)**. Images were adjusted for brightness and contrast. Scale bar = 50 μm. IBA-1-stained microglia were then converted to 8-bit, adjusted for brightness, and cleaned with a Gaussian filter. Images were converted to binary and skeletonized in ImageJ. Representative images of non-stressed WT, stressed WT, non-stressed Tg, and stressed Tg rats are shown **(B)**.

The estimated population of microglia in the hippocampus in females was unchanged by stress [*F*_(1,8)_ = 3.26, *p* > 0.05] or genotype [*F*_(1,8)_ = 0.32, *p* > 0.05, Table 5]. Hippocampal microglia in HIV-1 Tg rats exhibited an increased number of branches [*F*_(1,8)_ = 14.62, *p* < 0.01], number of junctions [*F*_(1,8)_ = 13.94, *p* < 0.01], and maximum branch length [*F*_(1,8)_ = 14.31, *p* < 0.01], but average branch length was unchanged [*F*_(1,8)_ = 1.45, *p* > 0.05] compared to WT rats (Figure 4). An *a priori t*-test showed that stress increased the number of branches (*t*_4_ = 4.64, *p* = 0.01) and number of junctions (*t*_4_ = 4.46, *p* = 0.01) in WT-stressed rats compared to WT controls. Representative images and skeletonized microglia are shown in Figure 5.

**Table 5:** **The estimated population of IBA-1-stained microglia was determined using the optical fractionator probe (Stereo Investigator)**.

		Mean (estimated number of cells)	SEM
Wild type	No stress	4.28 × 10^10^	5.29 × 10^9^
	Stress	3.95 × 10^10^	1.91 × 10^9^
HIV-1 transgenic	No stress	4.34 × 10^10^	5.72 × 10^8^
	Stress	3.53 × 10^10^	2.8 × 10^9^

*A two-way ANOVA was used to determine the statistical significance. There were no significant main effects of stress or HIV-1 genotype nor interactions to impact the estimated population of microglia. Mean and SEM are shown. α = 0.05*.

Estimated blood vessel length in WT and HIV-1 Tg rats was not significantly different in the PFC (*t*_6_ = 0.2436, *p* = 0.8157), hippocampus (*t*_9_ = 1.667, *p* = 0.1298), or amygdala (*t*_9_ = 0.2770, *p* = 0.7880). Blood vessel density was also unchanged in the PFC (*t*_6_ = 1.064, *p* > 0.05), hippocampus (*t*_9_ = 1.007, *p* > 0.05), and amygdala (*t*_9_ = 1.044, *p* > 0.05) (Table 6).

**Table 6:** **Blood vessel length and density were estimated using the Spaceballs probe (Stereo Investigator)**.

Region	Measure	Genotype	*n*	Mean ± SEM	*T* statistic	*p*-value
Hippocampus	Length (μm)	WT	5	5.653 × 10^6^ ± 439454	*t*_9_ = 1.667	0.1298
		Tg	6	4.743 × 10^6^ ± 339171
	Density (μm/μm^3^)	WT	5	2.025 ± 0.2465	*t*_9_ = 1.007	0.3401
		Tg	6	1.774 ± 0.1019
PFC	Length (μm)	WT	3	4.380 × 10^6^ ± 920262	*t*_6_ = 0.2436	0.8157
		Tg	5	4.633 × 10^6^ ± 595337
	Density (μm/μm^3^)	WT	3	2.551 ± 0.08156	*t*_6_ = 1.064	0.3281
		Tg	5	3.164 ± 0.4294
Amygdala	Length (μm)	WT	5	3.591 × 10^6^ ± 189420	*t*_9_ = 0.2770	0.788
		Tg	6	3.700 × 10^6^ ± 320519
	Density (μm/μm^3^)	WT	5	1.280 ± 0.07705	*t*_9_ = 1.044	0.3237
		Tg	6	1.402 ± 0.08481

*A student’s *t*-test was used to compare vessel length and density between WT and HIV-1 Tg rats in the hippocampus, PFC, and amygdala. Mean, SEM, and test statistics are shown. HIV-1 Tg rats did not exhibit significantly different blood vessel length or density in any region. α = 0.05*.

### Expression of Inflammatory Markers is Increased in Female HIV-1 Tg Rats

**Figure 6:**
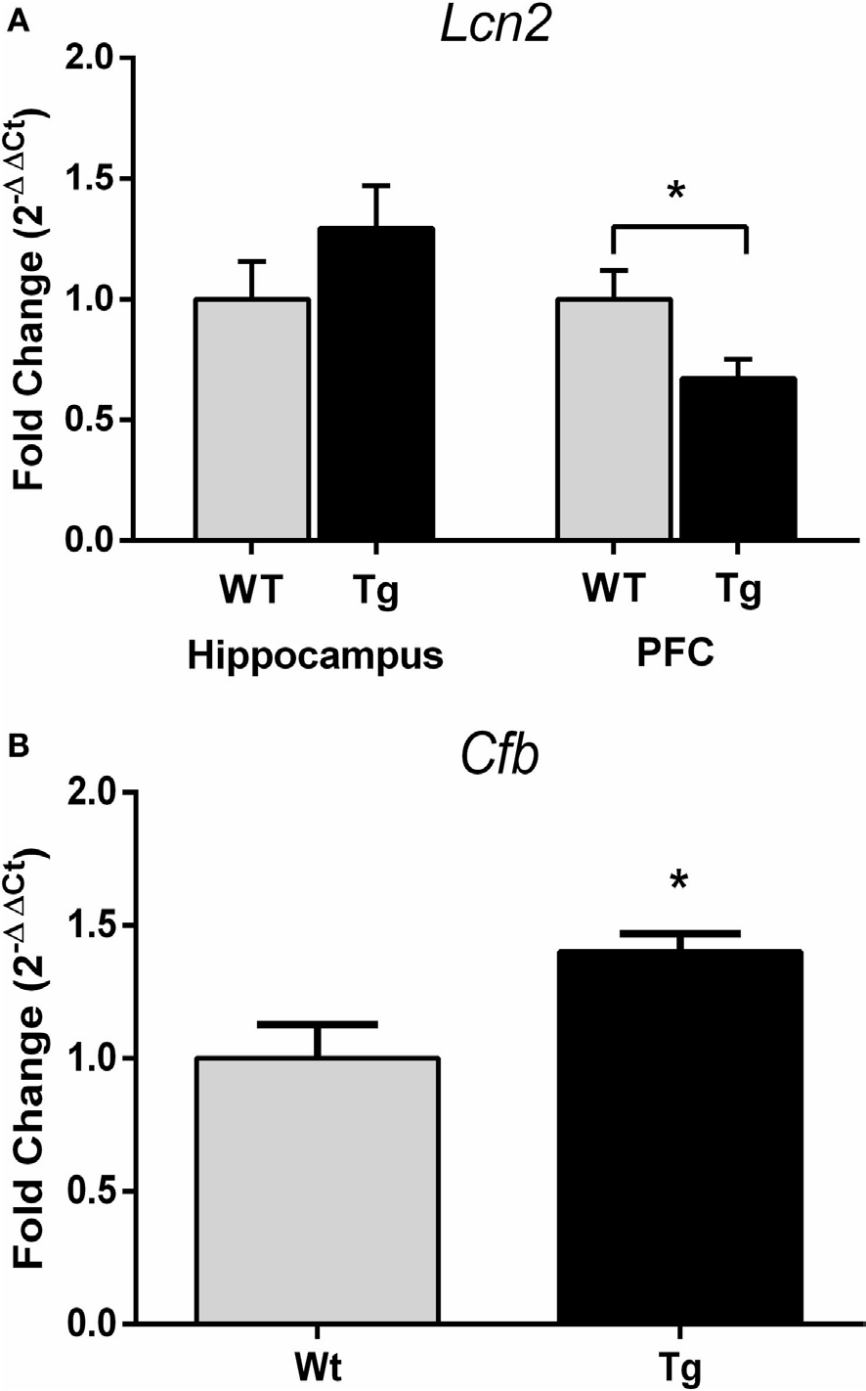
**Quantitative real-time PCR was performed to assess gene expression of complement factor B and lipocalin-2**. *Lcn2* expression was decreased in the prefrontal cortex but not hippocampus of HIV-1 Tg rats **(A)**. *Cfb* gene expression was increased in the hippocampus of HIV-1 Tg rats **(B)**. Data are presented as mean ± SEM. *Denotes a main effect of HIV-1 transgene. α = 0.05.

Gene expression of complement factor B (*Cfb*) in the hippocampus was higher in female HIV-1 Tg rats compared to WT rats (*t*_19_ = 2.87, *p* = 0.01) (Figure 6B). Gene expression of lipocalin-2 (*Lcn2*) was unchanged in the hippocampus (*t*_15_ = 1.18, *p* > 0.05) but decreased in the PFC (*t*_15_ = 2.40, *p* = 0.03) in female HIV-1 Tg rats compared to WT controls (Figure 6A). Statistical analysis information is detailed in Table 7.

**Table 7:** ***F*-statistics and *p*-values for all main effects and interactions are shown for microglia and gene expression analysis**.

	HIV-1 genotype	Stress	Stress × HIV-1 genotype
Microglial branches (4A)	*F_(1,8)_ = 14.62, *p* < 0.01	*F*_(1,8)_ = 2.17, *p* > 0.05	*F*_(1,8)_ = 2.38, *p* > 0.05
Microglial junctions (4B)	*F_(1,8)_ = 13.94, *p* < 0.01	*F*_(1,8)_ = 2.98, *p* > 0.05	*F*_(1,8)_ = 2.59, *p* > 0.05
Microglial max branch length (4C)	*F_(1,8)_ = 14.31, *p* < 0.01	*F*_(1,8)_ = 0.29, *p* > 0.05	*F*_(1,8)_ = 0.02, *p* > 0.05
Average branch length (4D)	*F*_(1,8)_ = 1.45, *p* > 0.05	*F*_(1,8)_ = 0.97, *p* > 0.05	*F*_(1,8)_ = 1.32, *p* > 0.05
*Lcn2* hippocampus (4A)	*t*_15_ = 1.18, *p* > 0.05		
*Lcn2* PFC (4A)	*t_15_ = 2.40, *p* = 0.03		
*Cfb* (4B)	*t_19_ = 2.87, *p* = 0.01		
IBA-1-estimated population (Table 5)	*F*_(1,8)_ = 0.32, *p* > 0.05	*F*_(1,8)_ = 3.26, *p* > 0.05	*F*_(1,8)_ = 0.54, *p* > 0.05

A two-way ANOVA was used for microglial morphology analysis (between-factors HIV-1 genotype and stress). A student’s *t*-test was used to assess significance of gene expression data. *Indicates a significant main effect or interaction. Blank boxes indicate an absence of a particular factor from analysis. The figure or table number is included in parentheses following the description of each metric. α = 0.05.

## Discussion

Collectively, the data presented here demonstrate that development in the presence of HIV-1 proteins leads to anxiety-like behavior and ramification of microglia. The combination of chronic adolescent stress with HIV-1 protein expression did not further exacerbate the impact of HIV-1 proteins in females. While the presence of HIV-1 proteins can alter male body morphology and behavior, the impact on these metrics appears to be more salient in adolescent females.

Although HIV-1 Tg female rats exhibited normal locomotor behavior during the dark cycle, during the more anxiety-provoking light cycle, female HIV-1 Tg rats exhibited decreased locomotor activity in the open field and reduced central tendency as compared to WT females. This context-specific change in locomotor activity suggests that the alterations in distance traveled are not due to a physical impairment. This is consistent with a previous study in adolescent female HIV-1 Tg rats in which a trend toward decreased time in the center of an open-field maze was observed (15). All rats, regardless of genotype, sex, or stress, showed reduced central tendency in the light cycle compared to the dark cycle, suggesting that all rats responded to the anxiety-provoking stimulus of bright light.

Unlike previous reports from our group that demonstrated an increase in anxiety-like behavior in female rats following chronic adolescent stress (21, 22, 28), the current study did not find a main effect of stress on central tendency in the open field. This may be a reflection of strain-specific sensitivities to chronic adolescent stress, as the previous reports from our group were conducted in Wistar or Sprague–Dawley rats (21, 22, 28), and the current study was conducted in Fisher rats as these are the background strain of the HIV-1 Tg rat. While the timing of the adolescent stress paradigm was consistent with previous studies from our group that documented behavioral effects of chronic adolescent stress (21), it is possible that, had the adolescent stress paradigm continued throughout the behavioral testing days, the behavioral effects could have been more robust. And while the adolescent stress paradigm takes place during the average age of pubertal onset, timing that may be particularly disruptive to female behavior, no behavioral effects of adolescent stress were observed in females or males. However, both chronic adolescent stress and the presence of HIV-1 viral proteins reduced adolescent weight gain (PND36–46). The fact that chronic stress was able to alter physiological endpoints suggests that although Fisher rats may not be as behaviorally sensitive to chronic adolescent stress as Wistar and Sprague–Dawley rats, chronic adolescent stress is a salient environmental stimuli in the Fisher strain.

The novel object recognition task was performed to assess object recognition memory. The test was performed with either no delay or 1-h delay following exposure to the familiar objects. The no-delay condition serves as a control for confounds of differences in investigative behavior that could be driven by an underlying difference in anxiety-like behavior. A non-zero difference in novel vs. familiar object investigation may be interpreted as lack of memory impairment; that is, the animal differentiates between the two objects and displays a preference for one. Both female and male HIV-1 Tg rats spent a decreased amount of time sniffing all objects in the no-delay paradigm as compared to WT controls. Female HIV-1 Tg rats displayed a reduction in investigation of novel and familiar objects compared to WT females. Though this reduction is present in both the no delay and 1-h delay conditions, rats persisted in displaying a difference in time spent exploring the novel vs. familiar objects, suggesting object recognition despite reduced overall exploration. Although a cognitive deficit cannot be completely ruled out, the combination of reduced investigative behavior and no difference between no-delay and hour-delay conditions suggests that these animals do not suffer from overt memory impairments, but rather a decrease in investigation. The current testing is not designed to determine if this decreased investigation is a reflection of decreased motivation or increased anxiety-like behavior; however, an interpretation of increased anxiety-like behavior is consistent with the anxiety-like behavior observed in the open-field test. Previous studies have reported mixed results regarding memory impairment in HIV-1 Tg rats. In the Morris Water Maze, HIV-1 Tg rats exhibited decreased learning but no change in memory of the hidden platform (29). Conversely, a study by Repunte-Canonigo et al. observed impaired working memory in the T-maze (16). Here, we had hypothesized that stress and HIV-1 proteins would interact to impair memory, but these data suggest that neither condition alone nor the combined impact of these factors is sufficient to cause memory impairment in adolescent rats.

In order to collect tissue at the same age and without the stress of estrous cycle tracking, samples from female rats could not be collected at every stage of the estrous cycle. Both the behavioral metrics (PND49–53) and the weight data were collected across multiple days and therefore could not be tied to any one stage of the estrous cycle. As we have reported previously (21, 22, 28), we collected uterine weights at the completion of the study to determine whether systematic group differences in estrous cycle might be present. Figure 1D demonstrates that uterine weights were not systematically different among groups, and therefore, there likely was not a group difference in estrous cycle stage. These data confirm that reported differences in behavior and inflammatory endpoints are likely not caused by different estrous cycle stages among groups; however, variations in estrous cycle stage may contribute to variability within groups, though a previous report suggests that differences in estrous cycle do not increase group variability over what is observed in males (30).

Our current findings of anxiety-like behavioral alterations in females are consistent with our previous demonstration of altered affective-like behavior and stress responses in female HIV-1 Tg rats (15, 31). In order to elucidate the mechanisms potentially underlying these behavioral changes, we evaluated microglial and vascular morphology in female HIV-1 Tg rats. The hippocampus has been previously investigated in HIV-1 Tg rats due to its established susceptibility to neuroinflammation and damage by HIV-1 viral protein exposure (32, 33), and we have previously reported HIV-1 induced deficits in hippocampal neurogenesis (15). In the present study, we first examined whether microglial activation, indicative of neuroinflammation, was altered with stress or in female HIV-1 Tg rats. The estimated population of IBA-1 positive microglia was unchanged with stress or exposure to HIV-1 transgene. While we did not observe differences in the number of IBA-1 positive microglia, a previous study found increased gene expression of *Iba-1* in adult HIV-1 Tg rats, though they did not specify the sex of the animals tested (16). Thus, there may be potential sex- or age-dependent variations in microglial profiles in the HIV-1 Tg rats.

For more in-depth assessment of microglia, we examined microglial morphology and branching. Microglia in HIV-1 Tg female rats exhibited a hyper-ramified structure, characterized by increased number of junctions and branches. Hyper-ramification has been hypothesized to be an intermediate stage in microglial activation (34), and hyper-ramified microglia have been associated with impaired working memory following stress (35); however, detailed behavioral consequences of enhanced microglial ramification are not well established (35). The enhanced microglial ramification observed in the hippocampus of the adolescent female HIV-1 Tg rats is likely a consequence of the presence of HIV-1 viral proteins because this morphology is present even in the absence of chronic stress exposure. However, it is premature to conclude that these changes in microglial morphology are behaviorally salient in this particular case. We observed similar ramification following chronic adolescent stress in female adolescent WT rats that did not co-occur with behavioral alterations. Collectively, these data suggest that both development with HIV-1 proteins and chronic adolescent stress are independently capable of inducing ramified microglia in the female adolescent hippocampus, but these two stimuli do not appear to interact at the level of microglial ramification.

In addition to microglial morphology, we assessed vascularization of the hippocampus based on previous demonstrations of altered vascularization following stress (36). There was no effect of HIV-1 on vascularization of the hippocampus (Table 1). Because of previous reports of alterations in the PFC of HIV-1 Tg rats (31) and the potential for stress to impact vascularization in other brain regions (36), we also assessed vascular endpoints in the PFC and amygdala. Similar to the hippocampus, vascularization of neither the PFC nor the amygdala was impacted by development in the presence of HIV-1 proteins. When these vascularization data are viewed in light of the alterations in microglial morphology reported above as well as previously published demonstration of reduced neurogenesis (15), they suggest that glial and neuronal alterations are not secondary to a primary change in vascular structure.

In order to determine if the inflammatory profile suggested by microglial ramification extended to other metrics, we assessed the expression of two inflammatory markers linked to HIV-1. We have previously reported that *Mcp-1* expression is elevated in the hippocampus of HIV-1 Tg female rats, but assessments of other portions of the innate immune system have not been reported (15). We assessed expression of complement factor B, a component of the alternative complement pathway in the innate immune system. Activity in the complement cascade in individuals with HIV-1 has previously been studied in context of HIV-associated cognitive impairments; previous studies found elevated protein expression of complement component 3 (C3) in patients diagnosed with HIV-associated dementia (37). Complement activation has also been investigated as a mechanism of neurodegeneration in patients with HIV-1 (38) as well as in other central nervous system disorders (39). As demonstrated in Figure 6, expression of *Cfb* was increased in the presence of HIV-1 proteins in adolescent female rats. An increase in activation of both the alternate and classical complement pathway in HIV patients has been reported (40), and it has been hypothesized that factor B is protective against HIV-1 infection (41). Interestingly, here, we saw increased complement factor B in the absence of a true infection as only the viral proteins are expressed, suggesting that the proteins themselves are an inflammatory stimulus.

The complement system has effects on neuronal architecture beyond the commonly appreciated inflammatory effects of this system (42), and these non-traditional impacts of innate immune system activation could contribute to impairments observed in PLWH. With this in mind, we also examined gene expression of lipocalin-2 (Lcn2), an acute phase protein that plays an important role in dendritic spine remodeling and anxiety-like behavior (43, 44), as dendritic damage has been cited as a contributor to development of HAND (45, 46). Although *Lcn2* expression was unaltered in the hippocampus, it was reduced in the PFC of HIV-1 Tg female rats. Region-specific alterations in gene expression have been reported previously in the HIV-1 Tg rat (31, 47). Further investigating inflammatory markers for their potential to effect stress- and HIV-1-induced changes in structural plasticity may help elucidate the mechanisms underlying the behavioral changes reported here.

Collectively, the current findings suggest that HIV-1 proteins are a potent physiologic and behavioral modifier. These findings reinforce the hypothesis that the biological impacts of HIV-1 viral protein exposure, rather than the psychosocial impacts of diagnosis and disease management, may be responsible for HIV-1-associated behavioral alterations. The use of a stress-based animal model provided the tools necessary to isolate the impact of psychosocial variables from biological influences of HIV-1 to gain insight into the mechanisms potentially underlying psychiatric and cognitive dysfunction in PLWH. Future studies should further elucidate specific mechanisms involved in HIV-1-related behavioral changes. The data presented here, in combination with previous reports (15), suggest that HIV-1 proteins disrupt behavior and lead to neuroinflammatory effects. However, the behavioral and neuroinflammatory effects are not directly linked, at least not by traditional mechanisms. Although HIV-1 is an immune disorder and has profound inflammatory implications, other systems may be more directly influential in behavioral alterations, such as the hypothalamic–pituitary–adrenal axis (31), neuronal morphology (42), or dopaminergic systems (13, 14).

## Author Contributions

GN generated the hypotheses and secured funding for the studies performed. CH designed the behavioral experiments in collaboration with GN and then performed the work. MB and SR designed the qPCR experiments in collaboration with GN and then performed the work. AG designed the microglial analysis plan under the supervision of GN and then performed the work. SK, MW, and RR designed the vascular analysis plan in consultation with GN and then performed the immunohistochemistry and vascular analysis. SK oversaw Tg rat care and performed adolescent stress. GN oversaw all analyses of the collected data. GN and SR wrote and edited the manuscript. All authors revised the manuscript.

## Conflict of Interest Statement

The authors declare that the research was conducted in the absence of any commercial or financial relationships that could be construed as a potential conflict of interest.
